# Supplementary material for: Undernutrition, food insecurity, and leprosy in North Gondar Zone, Ethiopia: A case-control study to identify infection risk factors associated with poverty
Source: PLoS Negl Trop Dis. 2021 Jun 24;15(6):e0009456. doi: 10.1371/journal.pntd.0009456 (PMC8277044; doi:10.1371/journal.pntd.0009456)
Supplement: Attachment — Submitted filename: Comments_PNTD-D-20-01634.pdf [file pntd.0009456.s001.pdf]

Overall comment

I'm grateful for the opportunity to review your manuscript entitled "Undernutrition, food insecurity, and leprosy in North Gondar Zone, Ethiopia: A case-control study to identify infection risk factors associated with poverty". The study addressed an interesting issue and could be informative especially with little evidence available on the issue in Ethiopia. The submitted manuscript unfortunately requires major revision before it is ready for publication, as it lacks methodological detail that would facilitate sound interpretation of the results and permit reproducibility of the study elsewhere (details below).

Major comments by section

Abstract

1. It is important for the abstract to explain the objective and design of the study and mention relevant details on participants i.e. number of participants (cases and controls), case to control ratio, age
2. The abstract need to briefly explain how cases were defined

Author summary

1. Page 3, line 56 , is it on leprosy transmission? The authors stated *"Although the study focuses on the leprosy burden in North Gondar Zone, Ethiopia… "* I don't think the focus of the study is on the burden of leprosy. Both sentences need to be revised according to the objective of the study.
2. This section need to be revised based on comments below

Methods

1. It is unclear how the authors ascertain controls. On page 6, 108 -109 It is stated *"Controls were adults without contact of known cases of leprosy and who resided in the same communities as the cases…..Individuals with previous leprosy infection and suspicious skin or nerve symptoms were excluded as controls"* Is this enough to ascertain controls? This sentence could further be expanded to include if any physical examination and/or any laboratory confirmations were conducted. This is important because it could have led to misclassification. In addition, it is important to describe how controls were recruited i.e. from facility or community?

2. Measurement of variables is not clearly stated. It is explained, to some extent, how MUAC was measured. However, measurement procedures for other anthropometric parameters i.e. height and weight should be explained in detail including if there is training of data collectors, measures taken to ensure data quality, technical error measurements during training (if calculated) and other procedures followed while measuring height and weight need to be clearly explained. Using a separate paragraph for each measurement (anthropometric, dietary and others) under the headline of variables/measurements would improve organization.

3. I found it difficult to link the results of the study with the USDA food security measurement tool the authors described in the data collection section. The collected food security variables doesn't seem to appropriately measure food security. Moreover, the authors might want to explain why they chose this specific tool to assess food security. On quick literature review, there are evidences of a valid household food insecurity access scale to assess food insecurity including seasonality in Ethiopia (such as Gebreyesus et al. BMC Nutrition 2015, 1:2 http://www.biomedcentral.com/bmcnutr/content/1/1/2 and Kabalo et al. BMC Nutrition (2019) 5:54 https://doi.org/10.1186/s40795-019-0323-6).

4. The sources of measurements (data collection tool) used to assess dietary habit is also not clear and it is very difficult to say the variables stated in table 2 measure dietary habit. Table 2; what does "recently modified dietary intake mean"? Is this increasing or decreasing dietary intake? By how much? How it was measured need to be clearly stated. Is it common to take dietary supplements in Ethiopia, particularly in the study setting?

5. The data analysis section mainly talks about sample size. It would be better understandable if sample size and data analysis were described as separate sections.

6. On page 7, line 147-148; It is stated *"education (found to be the most useful marker of socioeconomic status in our study)."* How did the authors reach to this conclusion? Also page 9 line 181 stated *"education (as a surrogate marker of socioeconomic status)".* These statements seem debatable considering it is stated, in the study setting section, that the vast majority of the population in the study area live in agricultural areas.

7. Why is it that socioeconomic variables i.e. wealth index or at least monthly family income were not measured?

Result

1. It looks like majority (75%) of the participants were males. This number is almost similar between cases and controls. Is this a coincidence or did the authors match cases and controls by sex? In case of the later, the authors need to consider appropriate analysis.

2. Results of a characteristics comparison between cases and controls is not included for each variable as stated in the analysis although there is a figure for MUAC and BMI. It would be easy to understand if p-vaues were written next to each variable embedded in table 1.

3. While classifying BMI, Normal and Obese individuals should not be in the same category.

4. Presenting the univariate and multivariate analysis results in one table, by clearly indicating the reference categories for each of the variables would improve understanding.

Some minor comments

1. Expand abbreviation in the abstract
2. Page 6, line 108*; "Other case inclusion criteria were being 18 years of age or older* ***or*** *residing in North Gondar Zone of Ethiopia"* is it if a person fulfils either one of the 2 criteria or both?
3. Page 6, line 114; was the demographic questions asked in a language other than Amharic?
4. Page 6, 119-21; write measurement unit next to the numbers
5. The sentence "*A p-value of < 0.05 was considered significant*." Is repeated on page 7 lines 143 and 150. I think it is enough to just keep one. Same goes for the analysis software.
6. Page 8, lines 63-65; the numbers in table 2 and sentences doesn't match.
