# Supplementary material for: Undernutrition, food insecurity, and leprosy in North Gondar Zone, Ethiopia: A case-control study to identify infection risk factors associated with poverty
Source: PLoS Negl Trop Dis. 2021 Jun 24;15(6):e0009456. doi: 10.1371/journal.pntd.0009456 (PMC8277044; doi:10.1371/journal.pntd.0009456)
Supplement: Attachment — Submitted filename: CommentsResponses_Revision_Final.docx [file pntd.0009456.s002.docx]

**Re: Manuscript: Undernutrition, food insecurity, and leprosy in North Gondar Zone, Ethiopia: A case-control study to identify infection risk factors associated with poverty**

**Response to Reviewers**

We thank the reviewers for their time and for providing constructive comments that significantly strengthened our manuscript. We have addressed all the comments by providing a point-by-point response detailed below.

**Overall comment**

*I’m grateful for the opportunity to review your manuscript entitled “Undernutrition, food insecurity, and leprosy in North Gondar Zone, Ethiopia: A case-control study to identify infection risk factors associated with poverty”. The study addressed an interesting issue and could be informative especially with little evidence available on the issue in Ethiopia. The submitted manuscript unfortunately requires major revision before it is ready for publication, as it lacks methodological detail that would facilitate sound interpretation of the results and permit reproducibility of the study elsewhere (details below).*

**Response:**

We would like to thank the Associate Editor and reviewers for their review of our submission and the many comments provided. We are grateful for the thorough feedback provided and hope that the revisions incorporated are well-received. The methods section has been improved, based on the reviewer comments, to enhance the interpretation of other sections of the manuscript. Please see below for detailed point-by-point responses, as requested.

**Major comments by section**

**Abstract**

1. *It is important for the abstract to explain the objective and design of the study and mention relevant details on participants i.e. number of participants (cases and controls), case to control ratio, age*

**Response:** Thank you for this comment. Participant characteristics are now better described in the results section of the abstract.

2. *The abstract need to briefly explain how cases were defined*

**Response:** Thank you for this point. Definition added in “Methods” section as “patients with clinical leprosy. …”. In terms of the abstract, we clarified this a little more, but did not go into a lot of details due to the word number restraints.

**Author summary**

1. *Page 3, line 56 , is it on leprosy transmission? The authors stated “Although the study focuses on the leprosy burden in North Gondar Zone, Ethiopia… ” I don’t think the focus of the study is on the burden of leprosy. Both sentences need to be revised according to the objective of the study.*

**Response:** Thank you for this important point. On line 56, the sentence now reads, “…on leprosy susceptibility.”And later in the paragraph now reads, “Although the study focuses on leprosy susceptibility, as it relates to undernutrition, in North Gondar Zone,…” Both sentences have been revised to match the objective of the study.

2. *This section need to be revised based on comments below*

**Response:** Thank you for this comment. Revisions based on comments below have been incorporated.

**Methods**

1. *It is unclear how the authors ascertain controls. On page 6, 108 -109 It is stated “Controls were adults without contact of known cases of leprosy and who resided in the same communities as the cases…..Individuals with previous leprosy infection and suspicious skin or nerve symptoms were excluded as controls” Is this enough to ascertain controls? This sentence could further be expanded to include if any physical examination and/or any laboratory confirmations were conducted. This is important because it could have led to misclassification. In addition, it is important to describe how controls were recruited i.e. from facility or community?*

**Response:** We appreciate the reviewer bringing up this point as misclassification is always a possibility in these situations. This comment has been addressed by incorporating a clarifying statement regarding physical examination and medical history. Additionally, a statement was added to mention where controls were being recruited from to support a previous statement saying that controls resided in the same communities as the cases.

2. *Measurement of variables is not clearly stated. It is explained, to some extent, how MUAC was measured. However, measurement procedures for other anthropometric parameters i.e. height and weight should be explained in detail including if there is training of data collectors, measures taken to ensure data quality, technical error measurements during training (if calculated) and other procedures followed while measuring height and weight need to be clearly explained. Using a separate paragraph for each measurement (anthropometric, dietary and others) under the headline of variables/measurements would improve organization.*

**Response:** We appreciate the reviewers bringing up this point. To organize this section of the manuscript better, we have incorporated a new header titled “Variables and Measurement” where we describe anthropometric and dietary measurements including definitions. In the *Data Collection* section, we have incorporated more specific measurement procedures and data quality initiatives addressed during the study.

3. *I found it difficult to link the results of the study with the USDA food security measurement tool the authors described in the data collection section. The collected food security variables doesn’t seem to appropriately measure food security. Moreover, the authors might want to explain why they chose this specific tool to assess food security. On quick literature review, there are evidences of a valid household food insecurity access scale to assess food insecurity including seasonality in Ethiopia (such as Gebreyesus et al. BMC Nutrition 2015, 1:2* [*http://www.biomedcentral.com/bmcnutr/content/1/1/2*](http://www.biomedcentral.com/bmcnutr/content/1/1/2) *and Kabalo et al. BMC Nutrition (2019) 5:54* [*https://doi.org/10.1186/s40795-019-0323-6*](https://doi.org/10.1186/s40795-019-0323-6)*).*

**Response:**  We used the 6 question USDA food security measurement tool which has a set of validated questions, and we therefore felt confident that we have been able to measure food security. We do recognize, however, that there may be other tools that are better for this context (the 2019 study you list above was published 2 years after we designed this study). We have actually already adapted the HFIAS in our subsequent studies.

4. *The sources of measurements (data collection tool) used to assess dietary habit is also not clear and it is very difficult to say the variables stated in table 2 measure dietary habit. Table 2; what does “recently modified dietary intake mean”? Is this increasing or decreasing dietary intake? By how much? How it was measured need to be clearly stated. Is it common to take dietary supplements in Ethiopia, particularly in the study setting?*

**Response:** Thank you for this comment, we realize that these questions are a bit unclear. And since we did not delve further into what they could mean, we went ahead and took these results out of Table 2.

5. *The data analysis section mainly talks about sample size. It would be better understandable if sample size and data analysis were described as separate sections.*

**Response:**  We have divided this section as recommended.

6. *On page 7, line 147-148; It is stated “education (found to be the most useful marker of socioeconomic status in our study).” How did the authors reach to this conclusion? Also page 9 line 181 stated “education (as a surrogate marker of socioeconomic status)“. These statements seem debatable considering it is stated, in the study setting section, that the vast majority of the population in the study area live in agricultural areas.*

**Response:** Thank you for this comment. Based on the recommendation, we have now removed the statement “…education (found to be the most useful marker of socioeconomic status in our study).” This conclusion was reached by the results of the study but recognize that it’s inclusion in the *Data Management & Analysis* section may be misleading. Although the vast majority of the population in the study area live in agricultural areas, some were traveling to health centers and nearby towns. Similarly, their education and education levels may not be limited or tied to the agricultural areas where they live.

7. *Why is it that socioeconomic variables i.e. wealth index or at least monthly family income were not measured?*

**Response:** Thank you for this comment. Although this was considered early on during the study design, after further discussions with colleagues who work in these regions, we were advised 1. Not to ask directly about income. 2. That many of the asset questions we did have did not seem to capture SES appropriately. So given the homogeneity of SES in this area (significant levels of poverty), we decided that education level would be a better and more reliable discriminator of resources / privilege, and thus used this as a confounder variable in the model.

**Result**

1. *It looks like majority (75%) of the participants were males. This number is almost similar between cases and controls. Is this a coincidence or did the authors match cases and controls by sex? In case of the later, the authors need to consider appropriate analysis.*

**Response:** Thank you for this comment. Excellent observation, however, this was coincidence. Cases and controls were not matched by sex.

2. *Results of a characteristics comparison between cases and controls is not included for each variable as stated in the analysis although there is a figure for MUAC and BMI. It would be easy to understand if p-vaues were written next to each variable embedded in table 1.*

**Response:** Thank you for this comment, we have added the p-values to table 1.

3. *While classifying BMI, Normal and Obese individuals should not be in the same category.*

**Response:** We wanted to identify underweight as the risk factor, and that is why we dichomitized the variable– furthermore, there was only 1 individual with a BMI in the obese category and 3 in overweight, so these were not sufficient numbers to categorize on their own. We have now included the overweight / obese category in Table 1 to better show this.

4. *Presenting the univariate and multivariate analysis results in one table, by clearly indicating the reference categories for each of the variables would improve understanding.*

**Response:** We have done this as recommended. We also found one misclassification error as we were doing this (a control was misclassified as underweight) so we have adjusted the results and rechecked to ensure no other errors.

**Some minor comments**

1. *Expand abbreviation in the abstract*

**Response:** Thank you for this comment. “MB” is now expanded for clarity.

2. *Page 6, line 108; “Other case inclusion criteria were being 18 years of age or older or residing in North Gondar Zone of Ethiopia” is it if a person fulfils either one of the 2 criteria or both?*

**Response:** We really appreciate the reviewers bringing this to our attention as it is a very significant distinction to make. Revision has been made to replace “or” with “and” as study participants met both criteria for enrollment.

3. *Page 6, line 114; was the demographic questions asked in a language other than Amharic?*

**Response:** Thank you for this comment. Demographic questions were only asked in Amharic. To clarify this interpretation, “…a questionnaire **administered only** in Amharic…” has been added to the manuscript.

4. *Page 6, 119-21; write measurement unit next to the numbers*

**Response:** Thank you for this comment. Measurement units have now been incorporated next to BMI values.

5. *The sentence “A p-value of < 0.05 was considered significant.” Is repeated on page 7 lines 143 and 150. I think it is enough to just keep one. Same goes for the analysis software.*

**Response:** Thank you for this comment. In agreement with the reviewers, the sentence on line 150 has been omitted. For the analysis aspect, I have kept the following text “*Result is significant with a p-value <0.05.”, to distinguish between significant results in the tables.

6. *Page 8, lines 63-65; the numbers in table 2 and sentences doesn’t match.*

**Response:** We have reviewed to ensure that the results section and Table 2 align.
